# Five-Year Longitudinal Assessment of the Downstream Impact on Schistosomiasis Transmission following Closure of the Three Gorges Dam

**DOI:** 10.1371/journal.pntd.0001588

**Published:** 2012-04-10

**Authors:** Darren J. Gray, Aaron P. Thrift, Gail M. Williams, Feng Zheng, Yue-Sheng Li, Jiagang Guo, Honggen Chen, Tianping Wang, Xin Jiang Xu, Rong Zhu, Hongqing Zhu, Chun Li Cao, Dan Dan Lin, Zhen Yuan Zhao, Robert S. Li, George M. Davis, Donald P. McManus

**Affiliations:** 1 Molecular Parasitology Laboratory, Infectious Diseases Division, Queensland Institute of Medical Research, Herston, Brisbane, Queensland, Australia; 2 Griffith Health Institute, Griffith University, Meadowbrook, Queensland, Australia; 3 School of Population Health, University of Queensland, Brisbane, Queensland, Australia; 4 Cancer Control Group, Queensland Institute of Medical Research, Brisbane, Queensland, Australia; 5 National Institute of Parasitic Disease, Chinese Centre for Disease Control and Prevention, Shanghai, People's Republic of China; 6 Hunan Institute of Parasitic Diseases, World Health Organization Collaborating Centre for Research and Control on Schistosomiasis in Lake Region, Yueyang, People's Republic of China; 7 Jiangxi Provincial Institute of Parasitic Diseases, Nanchang, People's Republic of China; 8 Anhui Institute for Schistosomiasis Control, Hefei, People's Republic of China; 9 Hubei Institute for Schistosomiasis Control, Wuhan, People's Republic of China; 10 Department of Microbiology and Tropical Medicine, George Washington University Medical Centre, Washington D.C., United States of America; London School of Hygiene and Tropical Medicine, United Kingdom

## Abstract

**Background:**

*Schistosoma japonicum* is a major public health concern in the Peoples' Republic of China (PRC), with about 800,000 people infected and another 50 million living in areas at risk of infection. Based on ecological, environmental, population genetic and molecular factors, schistosomiasis transmission in PRC can be categorised into four discrete ecosystems or transmission modes. It is predicted that, long-term, the Three Gorges Dam (TGD) will impact upon the transmission of schistosomiasis in the PRC, with varying degree across the four transmission modes.

**Methodology/Principal Findings:**

We undertook longitudinal surveillance from 2002 to 2006 in sentinel villages of the three transmission modes below the TGD across four provinces (Hunan, Jiangxi, Hubei and Anhui) to determine whether there was any immediate impact of the TGD on schistosomiasis transmission. Eight sentinel villages were selected to represent both province and transmission mode. The primary end point measured was human incidence. Here we present the results of this five-year longitudinal cohort study. Results showed that the incidence of human *S. japonicum* infection declined considerably within individual villages and overall mode over the course of the study. This is also reflected in the yearly odds ratios (adjusted) for infection risk that showed significant (P<0.01) downward trends in all modes over the follow-up period.

**Conclusions/Significance:**

The decrease in human *S. japonicum* incidence observed across all transmission modes in this study can probably be attributed to the annual human and bovine PZQ chemotherapy. If an increase in schistosome transmission had occurred as a result of the TGD, it would be of negligible size compared to the treatment induced decline seen here. It appears therefore that there has been virtually no immediate impact of the TGD on schistosomiasis transmission downstream of the dam.

## Author Summary

Schistosomiasis, caused by *Schistosoma japonicum*, is a significant parasitic disease and public health problem in China. How the parasite is transmitted there can be categorized into four distinct modes (modes I–IV) and it is predicted that the Three Gorges Dam, recently completed, will affect the way schistosomiasis is spread in these modes. We monitored transmission for a 5-year period (2002–2006) in eight villages, representative of the three modes (I–III) below the dam across four provinces (Hunan, Jiangxi, Hubei and Anhui) to determine whether there was any immediate impact of the dam on schistosomiasis spread. Human schistosomiasis incidence declined considerably within individual villages and each mode, and the yearly odds ratios (adjusted) for infection risk showed significant downward trends in all three modes over the follow-up period. The decreased human *S. japonicum* incidence recorded across transmission modes I–III was probably attributable to annual human and bovine praziquantel drug treatment. If an increase in schistosome transmission had occurred as a result of the dam, it would be of negligible size compared with this treatment-induced decline. There had thus been virtually no immediate impact of the TGD on schistosomiasis transmission downstream of the dam over the 5-year surveillance period.

## Introduction

**Figure 1:**
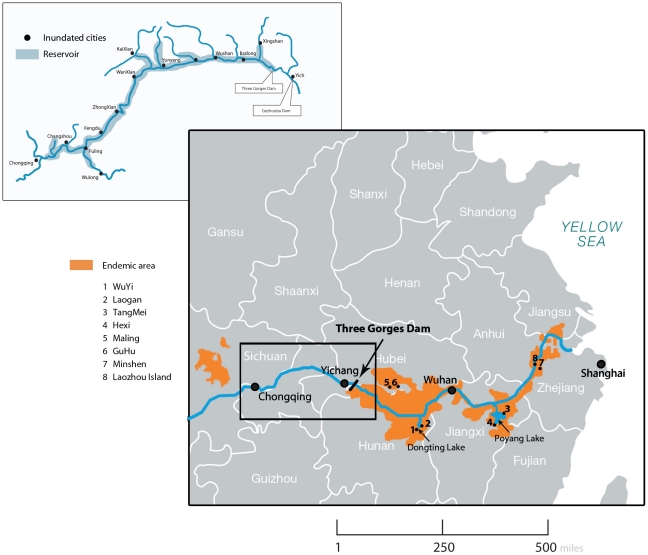
Three Gorges Dam and reservoir, distribution of *S. japonicum* and location of study villages.

Zoonotic schistosomiasis, caused by *Schistosoma japonicum*, is a chronic debilitating disease in the south of the People's Republic of China (PRC), with about 800,000 infected and 65 million people at risk of infection. [1], [2] The majority of transmission occurs in the lake and marshland areas of Jiangxi and Hunan, and in Jiangsu, Anhui and Hubei provinces; schistosomiasis is also endemic in the hilly and mountainous regions of Sichuan and Yunnan (Figure 1). [1], [3], [4] Based on ecological and environmental factors and *Oncomelania* snail population genetics, Davis et al. [3], [4] categorised schistosomiasis transmission in the PRC into four discrete ecosystems or ecogenetic transmission modes. The characteristics of modes I–III are presented in Table 1.

**Table 1:** Characteristics of *S. japonicum* transmission modes I–III* and predicted outcome on prevalence.

Mode	Environment	Provinces	Sentinel villages	Current characteristics	Long-term predicted outcome post-closure of the Three Gorges Dam
I	Poyang Lake	Jiangxi	TangMeiHexi	Poyang Lake is the largest lake in China and with the annual flood of the Yangtze River; the lake fills like a bathtub, covering most of the islands with water contained by man-made dikes constructed over centuries; and drowning snails. There is evidence for considerable transport of snails in the lake and they are considered to generally have an unstable population structure, although it is stable in some parts. Poyang Lake is a major focus of schistosomiasis transmission in PRC	The *Oncomelania* snail population structure will change from unstable to stable, large areas of marshland will not be flooded annually, and snail populations will increase considerably with a resultant increase in *S. japonicum* prevalence both in bovines and humans.
II	Dongting Lake	Hunan	WuYiLaogan	Dongting Lake plays an important role in regulating the amount of water in the Yangtze River. It collects the water of four rivers running from upstream into the Yangtze River and stores water when the Yangtze is in flood. It is a severe endemic area for *S. japonicum*	There will be increased permanent marshlands with increasing population stability, but in some areas near the Yangtze River there will be a continuing but somewhat decreased population instability associated with snail transport. Overall, snail populations will increase considerably with a resultant increase in *S. japonicum* prevalence both in bovines and humans.
	Yangtze River islands, flood plains	Anhui	Laozou IsMinshen	The Yangtze River islands and flood plains are covered and swept by the annual flood of the Yangtze River. On the islands, snails are found in low depressions in flat marshy grazing land. The flood plain habitat is a narrow strip of land along the river bounded by a high dike. The flood plain is moderately forested with small trees but has considerable grassland. These sites are annually covered and swept by the annual floods. Export and import of snails also occurs annually.	There will be continued snail transport and a continuing unstable snail population which will increase concomitant with an increase in *S. japonicum* prevalence in bovines and humans.
III	Canals, water networks	Hubei	MalingGuHu	Canals and water networks are protected from flooding from the Yangtze River by the continuous dike along the Yangtze and the water gate that is closed to the Yangtze during flooding	New snail habitats will be created due to the degeneration of rice paddies into marshlands as a result of underground water levels rising, thus increasing transmission and *S. japonicum* prevalence in bovines and humans

*: **After Davis et al.^2^**

The Three Gorges Dam (TGD) is one of several huge engineering projects transforming China's environment. [5] It is located in the Three Gorges region in the upper reaches of the Yangtze River (the world's third-largest river; 5920 km long) (Figure 1). It spans the Yangtze at Sandouping Island, just west of the city of Yichang in Hubei province. [1], [6], [7] The main justification for the dam is flood control; by regulating water flow it is designed to prevent disastrous floods that have occurred every decade along the lower plains regions of the Yangtze River. [1], [6], [7] Construction commenced in 1994 and by 2003 the TGD was closed to a height of 135 metres. In 2009, it reached its full height of 185 metres and its hydropower station began to generate 18,600MW of power. [8], [9] By 2009 the 2,300 m long dam had resulted in a 600 km long serpentine reservoir that inundated 115,000 acres of cultivated land, requiring the resettlement of some two million people [9] Long-term the dam is predicted to impact with varying degrees upon the transmission of schistosomiasis across the four transmission modes [6]–[8]; modes I–III are located downstream with mode IV upstream of the dam.

In mode I, it is predicted that long term the TGD will reduce annual flooding along the Yangtze, thus stabilising the populations of *Oncomelania* hupensis snails – the intermediate hosts of *S. japonicum* – resulting in increased transmission of schistosomiasis. In mode II, there will be increased permanent marshlands in some areas with increasing snail population stability; in other areas near the Yangtze River there is predicted to be continuing but somewhat decreased snail population instability associated with snail transport. Overall, in the long-term it is predicted that snail populations will increase considerably with a resultant increase in *S. japonicum* prevalence for mode II. In mode III, new snail habitats will be created due to the degeneration of rice paddies into marshlands as a result of underground water levels rising, with long-term predicted increased *S. japonicum* transmission. [7], [8]

We recently described the results [4] of 5-year longitudinal surveillance of *S. japonicum* transmission we undertook in Shian, in the Anning River Valley, a schistosomiasis-endemic village located upstream of the TGD in Sichuan province, typical of mode IV. The results of this study showed no effect on transmission over the study period [4], thereby corroborating our prediction of little or no immediate impact of the dam on schistosomiasis transmission in Sichuan. Nevertheless, we recommended continued surveillance as changes in transmission patterns may take upwards of 10 years to be realised as the water flow slows down and silt deposits settle, forming new marshland areas suitable for the propagation of *Oncomelania* snails. Snail dispersal and population movements will also be required to introduce schistosomes into this locality. [4]

Here we report the results of a similar study we undertook over the same period (2002–2006) in eight sentinel villages, representative of transmission modes I–III, downstream of the TGD located in Hunan, Jiangxi, Hubei and Anhui provinces.

## Methods

### Study design

We carried out a prospective longitudinal cohort study (2002–2006) in eight villages (Table 1), representative of schistosomiasis transmission modes I–III, to determine the potential impact of the closure of the TGD on schistosome incidence over time.

### Baseline

At baseline, two stool samples were collected and a questionnaire administered to all subjects usually resident in the eight study villages. Stool samples were examined microscopically using the Kato-Katz thick smear technique, with three slides per stool read blinded, to determine *S. japonicum* prevalence and intensity of infection. [10] The questionnaire consisted of questions relating to demographics, medical history and history of water contact. [11], [12]

A stool sample was also collected from all bovines (water buffaloes and cattle) in the study villages and examined for *S. japonicum* prevalence using the miracidial hatching test (3 individual hatches read blind; 50 grams of faeces per hatching) and intensity of infection, using a traditional Chinese sedimentation method. [11]

### Follow-up and study procedures

Following the baseline survey, a fixed cohort of all individuals aged 5–65 in each village was monitored for schistosome infection for the duration of the study. The cohort inclusion criteria were that an individual: a) must have been a resident of the village for more than 12 months; b) should be aged 5–65 years; c) did not intend migrating out of the village for the next 4 years; and d) should continuously reside in the village for the study period.

A water contact questionnaire, consisting of questions relating to each participant's yearly water exposure by season, was administered to all cohort members annually. [12]

Two stool samples were collected from all cohort subjects and one stool sample was obtained from all bovines to determine outcome measures; these included incidence and intensity of infection for cohort members, and infection rates and intensity of infection for bovines.

### Treatment regime

At baseline, all village residents and bovines found positive for *S. japonicum* were treated with praziquantel (PZQ) (humans: 40 mg/kg; bovines: 25 mg/kg), in accord with WHO recommendations, [11], [13] until the infection was cleared so that no fecal eggs were present. Our previous schistosomiasis studies around the Poyang and Dongting Lakes showed 85–95% efficacy for a single PZQ dose in humans and bovines, with 100% efficacy following re-examination and re-treatment. [13]–[16] At follow-up, all cohort members and all bovines found schistosome egg-positive were again treated with the same WHO recommended dose of PZQ until cleared of infection.

### Snail surveys

A snail survey was performed annually in April for each village to measure the prevalence of infection in snails and the density of infected snails per unit area. The survey used a Chinese random quadrat sampling method (0.11 metres^2^ sized frames, 20 metres between frames). [17]

### Water level readings

Water level readings were taken every 7–10 days from hydrological stations close to each village in modes I and II and directly measured in the canals within the villages in mode III. These were collected for the duration of the study (2002–2006).

### Data management and statistical analyses

A MS ACCESS based database was designed specifically for this project and was used for data management. [18] A positive human schistosome infection was indicated by the presence of at least one egg in any Kato-Katz smear. Egg counts were transformed to eggs per gram and geometric mean intensities were calculated using the log-transformed egg counts. Confidence intervals (CIs) were calculated using standard formulae based on the binomial distribution (annual incidence of infection) and the lognormal distribution (intensity). Each cohort member was assigned a water contact score for each year preceding infection status assessment. This was determined by adding season-specific sub-scores based on the frequency of water contact obtained through the water contact questionnaires. Formal analyses of annual human incidences, both crude and adjusted (for water contact, using the water contact score), used a generalized linear model (GLM) with a logit link and a binomial error distribution. Generalised equation estimators of parameters with an unstructured variance-covariance matrix were used to account for repeated measures on individuals over time. Analyses used the GENMOD procedure of SAS software (version 9.1; SAS Institute, Inc, Cary, NC) to calculate odds ratios (OR) and 95% confidence intervals (95% CIs).

## Results

### Baseline

#### Human prevalence and intensity of infection

The human *S. japonicum* prevalence (%) and intensity of infection (geometric mean eggs per gram (GMEPG)) within transmission modes I–III at baseline are shown in Table 2. The prevalence and infection intensity ranged from 13.5% (95% CI: 11.4–15.7) (N = 975) and 18.2 GMEPG (95% CI: 16.2–20.5) in mode III to 21.7% (95% CI: 19.3–24.2) (N = 1109) and 37.2 GMEPG (95% CI: 30.5–45.3) in mode I.

**Table 2:** Mode characteristics at baseline in 2002*.

Mode		I	II	III
**Human**				
	Sample size	1109	2310	975
	Prevalence	21.7% (19.3, 24.2)	14.0% (12.6, 15.4)	13.5% (11.4, 15.7)
	Geometric mean epg in infected humans	37.2 (30.5, 45.3)	30.3 (26.2, 34.8)	18.2 (16.2, 20.5)
	Sex ratio (F/M)	554/555	1078/1232	450/525
	Prevalence by sex (F/M)	19.9/23.6%	9.6/17.9%	14.0/13.1%
	Sentinel cohort no.	575	1129	354
	Sentinel cohort prevalence	21.6% (18.2, 24.9)	16.9% (14.7, 19.1)	14.1% (10.5, 17.8)
	Sentinel cohort geometric mean epg in infected humans	28.5 (22.1, 36.7)	32.6 (27.2, 39.0)	16.8 (13.6, 20.8)
	Sentinel cohort sex ratio (F/M)	307/268	536/593	166/188
	Sentinel cohort prevalence by sex (F/M)	19.5/23.9%	11.2/22.1%	13.9/14.4%
**Bovine**				
	Sample size	151	103	127
	Prevalence	11.9% (6.7, 17.1)	9.7% (3.9, 15.5)	13.4% (7.4, 19.4)
	Geometric mean epg in infected bovines	7.7 (3.6, 16.6)	9.6 (2.9, 31.8)	1.5 (1.2, 2.0)

*: Values in parentheses are 95% confidence intervals. epg = eggs per gram (of faeces).

Human prevalence and intensity of infection for the selected cohort at baseline within each sentinel village, representing transmission modes I–III, are shown in Table 3. Village prevalence ranged from 10.6% (95% CI: 8.2–12.9%) in Laogan (mode II) to 29.0% (95% CI: 25.5–32.4%) in Tang Mei (mode I). Infection intensity within the villages ranged from14.7 GMEPG (95% CI: 12.0–18.1) in Minshen (mode II) to 47.8 GMEPG (95% CI: 36.5–62.5) in Wu Yi (mode II).

**Table 3:** Human cohort infection rates and intensity of infection by study village over time.

Mode	Mode I		Mode II				Mode III	
Village	TangMei	Hexi	WuYi	Laogan	Laozhou Is	Minshen	Maling	GuHu
**Baseline**								
Sentinel cohort #	666	443	525	662	619	504	448	527
Prevalence (95% confidence interval; CI)	29.0% (25.5, 32.4)	10.8% (7.9, 13.7)	17.9% (14.6, 21.2)	10.6% (8.2, 12.9)	14.9% (12.1, 17.7)	13.5% (10.5, 16.5)	13.6% (10.4, 16.8)	13.5% (10.5, 16.4)
Geometric mean EPG (CI)	42.2 (33.5, 53.3)	22.2 (16.0, 30.8)	47.8 (36.5, 62.5)	19.3 (17.8, 20.9)	46.0 (33.4, 63.3)	14.7 (12.0, 18.1)	16.5 (14.1, 19.4)	19.9 (16.8, 23.6)
**2003**								
Sentinel cohort #	487	401	474	594	418	424	215	310
Incidence (CI)	23.8% (20.0, 27.6)	12.7% (9.4, 16.0)	11.4% (8.5, 14.3)	13.1% (10.4, 15.9)	13.4% (10.1, 16.7)	14.6% (11.2, 18.0)	14.4% (9.7, 19.2)	12.9% (9.2, 16.7)
Geometric mean EPG (CI)	34.7 (25.5, 47.3)	29.5 (21.9, 39.7)	45.6 (31.6, 65.8)	12.6 (10.2, 15.6)	109.7 (69.3,173.7)	35.1 (24.4, 50.6)	13.1 (9.2, 18.7)	8.7 (6.8, 11.1)
**2004**								
Sentinel cohort #	390	367	428	507	284	419	196	240
Incidence (CI)	18.7% (14.8, 22.6)	10.1% (7.0, 13.2)	12.6% (9.5, 15.8)	4.9% (3.0, 6.8)	10.2% (6.7, 13.8)	9.5% (6.7, 12.4)	7.1% (3.5, 10.8)	8.3% (4.8, 11.9)
Geometric mean EPG (CI)	30.1 (20.8, 43.6)	21.8 (17.2, 27.6)	51.3 (36.8, 71.5)	8.3 (5.9, 11.5)	205.2 (107.4, 392.0)	28.6 (20.0, 40.9)	13.6 (8.7, 21.3)	11.3 (8.8, 14.6)
**2005**								
Sentinel cohort #	358	348	379	422	263	319	174	269
Incidence (CI)	16.8% (12.9, 20.6)	9.8% (6.6, 12.9)	11.3% (8.1, 14.6)	5.7% (3.5, 7.9)	3.4% (1.2, 5.6)	8.8% (5.7, 11.9)	9.2% (4.9, 13.5)	9.7% (6.1, 13.2)
Geometric mean EPG (CI)	28.3 (19.1, 41.7)	22.9 (18.3, 28.6)	27.9 (21.1, 36.9)	23.8 (11.8, 48.1)	35.1 (24.2, 51.0)	22.7 (17.4, 29.6)	7.8 (4.6, 13.2)	6.9 (5.6, 8.6)
**2006**								
Sentinel cohort #	351	306	304	348	209	275	159	247
Incidence (CI)	6.0% (3.5, 8.5)	3.9% (1.7, 6.1)	8.2% (5.1, 11.3)	3.2% (1.3, 5.0)	1.4% (0.2, 3.1)	9.1% (5.7, 12.5)	5.0% (1.6, 8.5)	8.1% (4.7, 11.5)
Geometric mean EPG (CI)	25.2 (12.4, 51.4)	25.4 (12.9, 49.9)	22.3 (12.7, 39.2)	21.4 (8.1, 57.0)	193.8 (48.8,770.5)	28.2 (21.7, 36.6)	10.4 (4.6, 23.6)	12.5 (8.5, 18.6)

#### Human prevalence by sex and age

There were more males than females across the representative villages of the 3 transmission modes except those in mode III. Furthermore, except for the mode III villages, the baseline *S. japonicum* prevalence was higher in males than females (Table 2).

**Figure 2:**
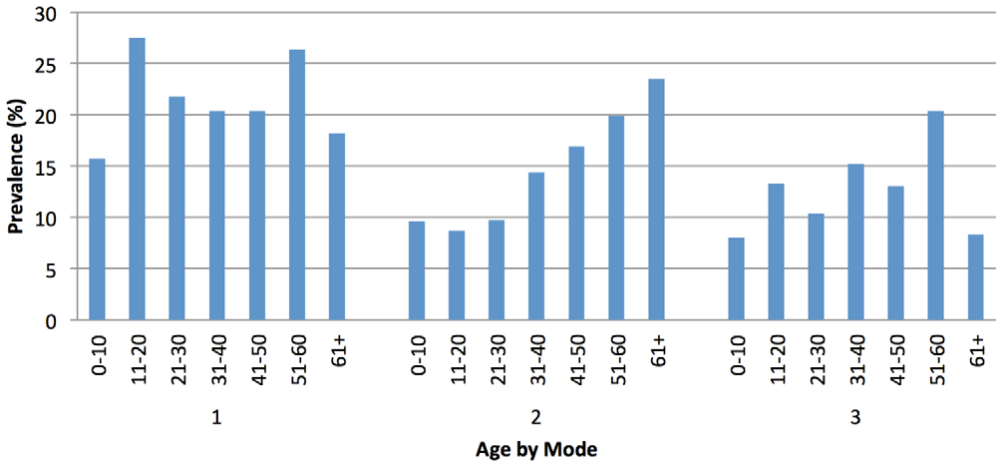
Baseline prevalence by age for transmission modes I–III.

*S. japonicum* prevalence by age varied across the transmission modes (Figure 2). Mode I had the highest prevalence in the age groups 11–20 and 51–60, mode II had the highest prevalence in the oldest age group (61+), and mode III had the highest prevalence in the 51–60 age group.

#### Bovine prevalence and intensity of infection

The *S. japonicum* prevalence (%) and intensity of infection (geometric mean eggs per gram (GMEPG)) in bovines at baseline showed that the mode III villages had the highest prevalence, but the lowest infection intensity (Table 2).

### Follow-up

#### Participant flow

The cohort numbers selected following baseline and the participant flow over the 4 years of follow-up is shown in Table 3. Loss to follow-up (mainly due to inhabitants leaving the villages to seek work in urban areas) per year for each village ranged generally from 2.0% to 24.0%, although some outliers were apparent - 27% in year 1 for Tang Mei; 33% and 32%, respectively, in years 1 and 2 for Laozhou Island; 41% in year 1 for Maling; and 52% in year 1 for Minshen. There was a 12% increase in the cohort in Gu Hu in year 4, due to the return of cohort-selected individuals who had left the village. Human PZQ treatment coverage was high with 98–100% of those found infected successfully treated.

#### Human incidence and intensity of infection

Over the four years of follow-up, the incidence of *S. japonicum* infection declined in all villages (Table 3) and in all three transmission modes (Table 4). Overall, reductions in incidence were 73.4% in mode I, 57.3% in mode II, and 48.9% in mode III (Table 4). The intensity of infection decreased within transmission modes I and II but not mode III. Infection intensity fluctuated within villages over the course of the trial, with 4 villages having a higher intensity of infection in 2006 than in 2003, especially those of mode II (Table 3).

**Table 4:** Human cohort incidence and intensity of infection by year for transmission modes I–III*.

Mode	I		II		III	
Human: sentinel cohort	N	Incidence	N	Incidence	N	Incidence
2003	888	18.8% (16.2,21.4)	1910	13.1% (11.6,14.6)	525	13.5% (10.6,16.5)
2004	757	14.5% (12.0,17.0)	1638	9.0% (7.6,10.4)	436	7.8% (5.3,10.3)
2005	706	13.3% (10.8,15.8)	1383	7.5% (6.1,8.9)	443	9.5% (6.7,12.2)
2006	657	5.0% (3.3,6.7)	1136	5.6% (4.3,7.0)	406	6.9% (4.4,9.4)

Human: sentinel cohort	N	Intensity (GMEPG)	N	Intensity (GMEPG)	N	Intensity (GMEPG)
2003	167	33.0 (26.2,41.6)	250	34.9 (28.7,42.4)	71	10.4 (8.4,12.8)
2004	110	27.0 (20.9,34.9)	148	42.2 (32.6,54.7)	34	12.2 (9.8,15.3)
2005	94	26.2 (20.2,33.9)	104	26.0 (21.2,31.9)	42	7.3 (5.8,9.1)
2006	33	25.3 (15.5,41.3)	64	26.9 (20.0,36.2)	28	11.9 (8.5,16.6)

*: Values in parentheses are 95% confidence intervals.

**Figure 3:**
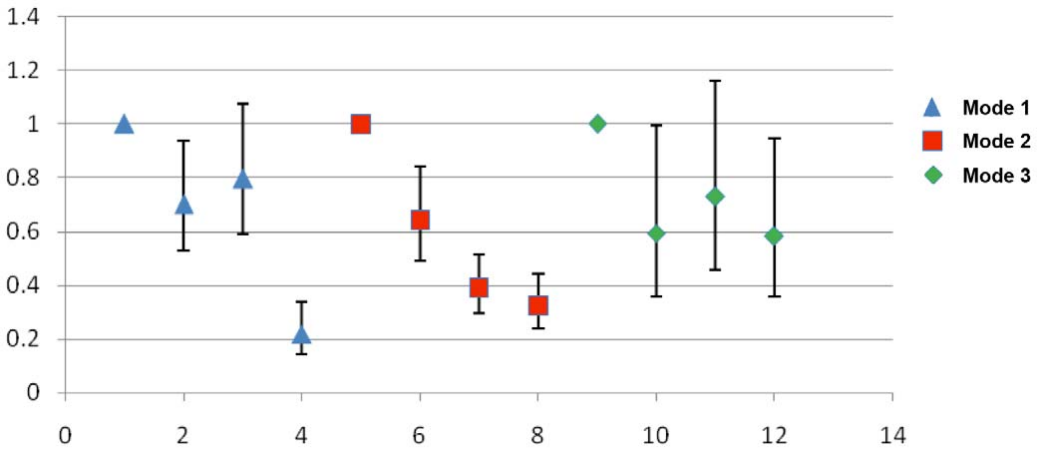
Annual risk (Odds ratios with 95% CI) of *S. japonicum* infection for transmission modes I–III. The model includes transmission mode, study year, previous praziquantel treatments, water contact, and an interaction term for transmission mode and year.

Regression analyses, yielding odds ratios (OR) adjusted for water contact for each transmission mode over the duration of the study (Figure 3), showed an overall significant (P<0.01) downward trend in infection risk across the three transmission modes in accord with *S. japonicum* incidence (Table 4). However, unlike transmission modes I and II, mode III exhibited an initial decrease in the risk of infection, followed by a flattening out of the trend such that the infection risk was higher than in the other two transmission modes. The reduction in infection risk was significantly different in each of the three modes (Figure 3).

#### Bovine infection rates and intensity of infection

The *S. japonicum* infection rates for bovines declined over the four years of follow-up in the transmission mode I villages (Table 5). The infection rates fluctuated in transmission modes II and III over the duration of the study with an overall decline in the latter, with no relative change in the former (Table 5). The intensity of infection also fluctuated over the follow-up period but there was an overall decline in transmission modes I and II but no change in mode III (Table 5).

**Table 5:** Bovine infection rates and intensity of infection by year for transmission modes I–III*.

Mode	I		II		III	
Bovines	N	Infection rate	N	Infection rate	N	Infection rate
2003	131	9.2% (4.2, 14.2)	165	17.0% (11.2, 22.8)	98	21.4% (13.2, 29.7)
2004	141	8.5% (3.8, 13.2)	157	9.6% (4.9, 14.2)	81	16.0% (7.9, 24.2)
2005	141	8.5% (3.8, 13.2)	134	14.2% (8.2, 20.2)	87	21.8% (13.0, 30.7)
2006	149	6.0% (2.2, 9.9)	95	17.9% (10.0, 25.7)	94	7.4% (2.0, 12.9)

Bovines	N	Intensity (GMEPG)	N	Intensity (GMEPG)	N	Intensity (GMEPG)
2003	12	6 (3, 10)	18	3 (2, 4)	21	8 (6, 10)
2004	12	5 (3, 8)	15	3 (2, 4)	9	7 (4, 11)
2005	12	2 (1, 3)	18	5 (3, 8)	17	21 (14, 31)
2006	9	2 (1, 3)	16	2 (2, 3)	7	8 (5, 11)

*: Values in parentheses are 95% confidence intervals.

### Dynamics of infected snails

The prevalence and density of infected snails fluctuated substantially over the five year study period and consistent trends could not be determined for any of the transmission modes. This was probably because of the high levels of snail sampling variability due to spatial aggregation effects that we have observed previously. [19]

### Water levels

Water level patterns in transmission modes I and II were similar over the duration of the study with peak levels in the summer coinciding with the rainy season. The levels in mode III fluctuated by about 50 cm around the maximum height of the canal systems in Maling and Guhu over the course of the study.

## Discussion

We describe the results of 5-year (2002–2006) longitudinal surveillance in eight sentinel villages representative of modes I–III of the four *S. japonicum* transmission modes described by Davis et al. [3] These villages are located downstream of the TGD in four provinces (Hunan, Jiangxi, Hubei and Anhui) and our aim was to determine whether there was any impact on *S. japonicum* transmission following closure of the dam and the commencement in 2003 of filling of the Three Gorges reservoir. [6]

The incidence of human *S. japonicum* infection declined considerably in all surveyed villages over the course of the study (Tables 3, 4). This was also reflected in the yearly adjusted odds ratios for infection risk which indicated significant (P<0.01) downward trends in all three transmission modes over the follow-up period (Figure 3). Regression analyses also showed that modal trends were significantly different from one another, thus indicating that the degree of decline in each mode was heterogeneous. The greatest decline was in mode I (73.4%), followed by modes II (57.3%) and III (48.9%).

The decrease in human incidence observed in transmission modes I–III may be attributable to the annual PZQ treatment, which on ethical grounds, had to be administered to all infected individuals. Major flooding of the lakes and marshland areas downstream of the TGD can drown adult snails resulting in decreased transmission, [1] but the water level records indicated that no major flood event had occurred during the study period. A number of reports have shown that bovines are the major transmission source for human schistosomiasis in the lakes and marshland areas of Southern China, and that interventions targeting bovines can reduce the incidence of human infection. [20]–[22] Over the follow-up period (2003–2006), bovine infection rates decreased in transmission modes I and III, but remained relatively stable for mode II, whereas infection intensity decreased in transmission modes I and II, but remained stable for mode III.

Therefore, the decrease in human *S. japonicum* incidence observed across the three transmission modes can probably be attributed to the annual human and bovine PZQ treatment. The differences in the downward trends evident between modes I–III may be due to the varying declines in bovine infection rates and infection intensity. Mode I had the greatest downward trend and declines in both bovine infection and intensity of infection, Mode II showed a decline only in bovine infection intensity, and whereas the infection rate declined in mode III, the infection intensity remained stable. It is noteworthy that there was no decrease in human exposure as water contact patterns did not change over the duration of the study, indicated by the similarity in crude and adjusted (for water contact) ORs. If an increase in schistosome transmission had occurred as a result of the TGD, it would have been negligible compared with the treatment-induced decline we observed. It appears, therefore, that there had been no or only very limited impact of the TGD on schistosomiasis transmission downstream of the dam over the 2002–2006 study period.

It is well recognised that schistosomiasis emergence or re-emergence has resulted following other large-scale hydro-projects such as the Gezira-Managil Dam in Sudan, the Aswan Dam in Egypt, the Melkasadi Dam in Ethiopia, and the Danling and Huangshi Dams in China. [23]–[26] It has been predicted that the TGD will alter water and sand distributions downstream that will have a significant impact on ecological systems; these include the Dongting and Poyang lakes and the canals of Hubei, where *S. japonicum* transmission is generally projected to increase, although decreased transmission is projected for other locations. [1], [6], [23], [24]

Specifically, it is anticipated that the TGD will result in large changes to the flow, depth and sedimentation load of the Yangtze so that the distribution and numbers of schistosome-infected *Oncomelania* snails will be altered, increasing transmission of schistosomiasis in some areas and its re-introduction into others where the infection is currently under control. [1], [5]–[8], [23], [24] Mathematical modelling has suggested a marked elevation in snail-breeding areas, increased infection rates of *S. japonicum* and greater associated morbidity. [7], [16] Seto and colleagues [6] believe that the lower more stable water levels downstream created by the TGD will result in decreased overall snail densities, but that the density of infected snails and corresponding human infections may increase due to the co-location of bovine grazing areas, snail habitats, and human activity that may occur with the more stable water levels. [1], [6]

The “Return Land to Lake” program currently underway in the PRC will significantly extend the water surface area in Dongting and Poyang Lakes, with the result that large numbers of farmers and fishermen are being resettled closer to lake water and *Oncomelania* snail habitats. [1], [24] This will also likely impact on schistosomiasis transmission as water contact and the prevalence and intensity of infection will increase. [1], [25], [27] Another important consideration is that the TGD reservoir, which has submerged wholly or in part 13 cities and 466 towns, has displaced up to 2 million people [5], [9] from non-endemic schistosomiasis localities upstream of the dam. Many of these villagers, having been relocated to downstream schistosome-endemic areas near lakes and wetlands in the Yangtze River Basin, will have no immunity to schistosomiasis, and hence will readily acquire a schistosome infection on exposure, and likely develop severe disease as a result.

Whilst no immediate effect of the TGD on schistosome transmission was evident in this study it may be that the predicted changes will take longer to eventuate. The dam, with its 1080 km^2^ reservoir, reached its full height in 2009 and this may be the critical time point that marks the start of environmental changes that will begin to impact on *S. japonicum* transmission. Continued surveillance should be undertaken to monitor the future ecological impacts of the dam. [28], [29] Accordingly, we have commenced a new study to monitor environmental changes and undertake longitudinal surveillance (2010–2014) of infection rates and intensity of *S. japonicum* infection in snails, humans and bovines in a further eight villages below the TGD to determine its effect on schistosome transmission dynamics. Findings from the study will be of considerable relevance for the PRC and other settings where schistosomiasis is endemic and where large water resource development projects are planned or are underway.

## Supporting Information

Checklist S1 (DOC)
