# Correction: Five-Year Longitudinal Assessment of the Downstream Impact on Schistosomiasis Transmission following Closure of the Three Gorges Dam

**DOI:** 10.1371/annotation/d78f7215-7e4e-4db3-ab80-66f44a101fdf

**Published:** 2012-06-06

**Authors:** Darren J. Gray, Aaron P. Thrift, Gail M. Williams, Feng Zheng, Yue-Sheng Li, Jiagang Guo, Honggen Chen, Tianping Wang, Xin Jiang Xu, Rong Zhu, Hongqing Zhu, Chun Li Cao, Dan Dan Lin, Zhen Yuan Zhao, Robert S. Li, George M. Davis, Donald P. McManus

The affiliation for the last author was incorrectly assigned. Donald P. McManus is not affiliated with #2 but with #1, Molecular Parasitology Laboratory, Infectious Diseases Division, Queensland Institute of Medical Research, Herston, Brisbane, Queensland, Australia. 

